# WT1 Expression Is Associated with Poor Overall Survival after Azacytidine and DLI in a Cohort of Adult AML and MDS Patients

**DOI:** 10.3390/cancers16173070

**Published:** 2024-09-04

**Authors:** Semra Aydin, Jennifer Schmitz, Chiara M. Dellacasa, Irene Dogliotti, Luisa Giaccone, Alessandro Busca

**Affiliations:** 1 Department of Oncology, Hematology, Immuno-Oncology and Rheumatology, University Hospital of Bonn, 53127 Bonn, Germany; 2 Division of Hematology, Department of Oncology, AOU Città della Salute e della Scienza di Torino, 10126 Turin, Italy; 3 Institute of Medical Biometry, Informatics and Epidemiology, University Hospital Bonn, 53127 Bonn, Germany; jschmitz@imbie.uni-bonn.de; 4 Stem Cell Transplant Center, Citta della Salute e della Scienza di Torino, 10126 Turin, Italy; cdellacasa@cittadellasalute.to.it (C.M.D.); abusca@cittadellasalute.to.it (A.B.); 5 Stem Cell Transplant Center, Hematology U, Città della Salute e della Scienza Turin, 10126 Turin, Italy; idogliotti@cittadellasalute.to.it (I.D.); luisa.giaccone@unito.it (L.G.); 6 Department of Molecular Biotechnology and Health Sciences, University of Turin, 10126 Turin, Italy

**Keywords:** acute myeloid leukemia, post-transplant relapse, Wilms tumor gene 1, azacytidine, donor lymphocyte infusions

## Abstract

**Simple Summary:**

Azacytidine and donor lymphocyte infusions were combined effectively in a single-center high-risk patient cohort (*n* = 29) for post-transplant relapse of acute myeloid leukemia and myelodysplastic syndrome. Bone marrow Wilms tumor gene 1 (WT1) expression was measured at time of transplant and post-transplant relapse. By using the Cox proportional hazards model, cut-off values for WT1 at both time points were obtained. The disease risk index incorporating initial cytogenetics and the disease stage at transplant as well as WT1 expression was significantly associated with overall survival, indicating that WT1 may represent a minimal residual disease marker in this context of cell therapy response evaluation, especially in high-risk patients currently considered to be in complete remission. Cut-off values for bone marrow WT1 expression for the two decisive treatment time points were proposed.

**Abstract:**

**Introduction:** Post-transplant relapse of acute myeloid leukemia and myelodysplastic syndrome faces restricted effective salvage regimens. We retrospectively analyzed the use of Azacitidine–donor lymphocyte infusion (AZA/DLI) in this setting. Furthermore, data on bone marrow Wilms tumor gene 1 (WT1) expression were collected. **Methods:** A Cox proportional hazards model, an outcome-oriented approach for the lowest smoothed plot of the martingale residuals, was performed for the cut-point determination of the respective WT1 expression levels. Finally, a Cox proportional hazards model investigated the association of overall survival (OS) with predictors. **Results:** An overall response of 41.4% with a median duration of 11.9 months for stable disease and 19.5 months for complete response (CR) patients was achieved. The disease risk index (DRI) high-/very high-risk patients had a shorter OS of 4.4 months than intermediate-risk patients, with 14.5 months, *p* = 0.007. At transplant, WT1-overexpressing patients (>150 copies) had a shorter median OS of 5.3 months than low-WT1-expressing ones, with 13.5 months, *p* = 0.024. Furthermore, patients with ≤1000 WT1 copies at relapse had a significantly longer OS with 15.3 months than patients overexpressing WT1, with 4.4 months, *p* = 0.0002. **Conclusions:** DRI and WT1 expression associate significantly with OS after AZA/DLI. Hence, WT1 may represent an MRD marker, especially in CR patients at high risk.

## 1. Introduction

Relapse of acute myeloid leukemia (AML) and myelodysplastic syndrome (MDS) after allogeneic hematopoietic stem cell transplantation (HSCT) displays 2-year survival rates of about 15% [1]. Proposed therapeutic options in this setting range from best supportive care, re-challenge with (intensive) chemotherapy and cellular approaches involving donor lymphocyte infusions (DLIs). The cumulative toxicity of the preceding HSCT and eventual co-existing graft-versus-host disease (GVHD) make only low-toxic regimens feasible. Hence, treatment strategies mainly aim to reduce the disease burden and to enforce the graft-versus-leukemia (GVL) effect. For this purpose, 5-Azacytidine (AZA) was combined with DLI, showing initial feasibility and efficacy in this setting. Except a large German retrospective multicenter study [2], most published data are reported on smaller cohorts [3,4,5,6]. Beyond its direct cytotoxic effect, AZA increases the immunogenicity of AML blasts by activating genes encoding for MHC class I and tumor antigens [7,8,9]. Transferred to in vivo models, these immunomodulatory properties of AZA combined with DLI seem to reduce GVHD [10,11,12], hence inducing remissions in chemotherapy refractory patients.

Wilms tumor gene 1 (WT1) is overexpressed in AML, and several in vitro studies have shown the modulation of leukemic cell growth by the up- and downregulation of WT1 [13,14,15]. WT1 re-expression at the time of relapse indicates a pathophysiological role of the gene in leukemic survival. Recent in vitro studies imply even a role in MDS pathogenesis, showing that Azacytidine also reduces pro-inflammatory cytokine production of mesenchymal stem cells [16]. Therefore, WT1 has been proposed for minimal residual disease (MRD) assessment in the allogeneic HSCT setting as well as in patients where molecular aberrations suitable for sensitive MRD testing are absent or instable [17,18,19]. On the basis of these data, the hypothesis arises that WT1 expression may not only function as an MRD marker but also be related to post-transplant relapse treatment outcomes, especially when cellular treatment strategies are applied.

In the present study, we performed a retrospective analysis of AZA ± DLI treatment in a particularly high-risk patient cohort with the first relapse of AML and MDS after allogeneic HSCT. Its aim was to investigate the association of patient- and disease-related variables with overall survival (OS). In particular, the association between treatment response and bone marrow (BM) WT1 expression at two decisive treatment time points, namely at time of transplant and at time of post-transplant relapse, was investigated.

## 2. Patients and Treatment

A total of 29 clinically and molecularly well-characterized adult AML and MDS patients were treated at the Stem Cell Transplant Unit in Turin. 5-Azacytidine with or without DLI support was given for post-transplant relapse. Chemotherapy regimens other than 5-Azacytidine were excluded from the analysis. Hematologic relapses were defined according to ELN criteria [20]. Of note, complete reconstitution of hematopoiesis was not required for the definition of complete remission (CR) if cytopenia was caused by factors other than leukemia specific therapy, such as GVHD or CMV/EBV virus reactivation and/or antiviral treatment. Chimerism was assessed via the polymerase chain reaction of polymorphic microsatellite regions [21]. Since the use of AZA at time of the study was off-label according to the indications authorized in Italy for the drug, each clinical case was reviewed by the Hospitals Commissions for Off-Label Use of Drugs, which assessed the benefit–risk balance and licensed the treatment of each patient. According to Italian law (Italian Drugs Agency-AIFA, Guidelines for Observational Studies, 20 March 2008), no formal Institutional Ethics Committee (IEC) approval was required. The Italian law does not require the Ethics Committee to express an opinion on off-label drugs; therefore, the EC has no jurisdiction in this regard. Before starting AZA/DLI, personal informed consent for off-label treatment was signed by each patient.

5-Azacytidine was administered mainly in an out-patient setting at a dose of 75 mg/m^2^ from day 1 to 7 (*n* = 21) or a dose of 100 mg from day 1 to 5 (*n* = 8) every 28 days according to the individual decision of the treating physician. The decision for dose reduction to 100 mg day 1–5 was mainly in order to decrease treatment toxicity in frail patients displaying significant post-transplant toxicity. Donor lymphocyte infusions were infused at day 8 of every second cycle of AZA starting from the second cycle. In case of matched sibling donors, the DLI dosage started from 1 × 10^7^ CD3+cells/kg and escalated every second cycle to a maximum of 1 × 10^8^ CD3+cells/kg. In matched unrelated donors, DLIs were escalated from initially 1 × 10^6^ CD3+cells/kg to a maximum of 1 × 10^8^ CD3+cells/kg. In haplo-transplants, DLIs escalated from 1 × 10^4^ CD3+cells/kg to 5 × 10^7^ CD3+cells/kg. After 4 cycles, the response was evaluated using BM analysis. On the basis of the response and tolerance, treatment continued until relapse or disease progression. Donor lymphocyte infusions were withheld in the case of GVHD appearance. Disease Risk Index (DRI) stratification of the patients was conducted according to Armand et al. [22]. Bone marrow WT1 expression was determined as previously described [23] and available before transplant and at relapse in all patients.

## 3. Statistical Analysis

Overall survival (OS) was defined as the time from start of Azacytidine treatment until the date of death or last observation and estimated using the Kaplan–Meier method. Overall survival was compared between different subgroups using a two-sided log-rank test at a level of 5%. Quartile OS times were indicated with 95% confidence intervals for the respective subgroups. Regarding disease state at HSCT, patients with CR1 or CR > 1 were pooled to “CR/CR > 1”, while patients who had a stable disease (SD) or progressed were classified as “SD/progressive”. Regarding the DRI, very high-risk and high-risk patients were pooled together and compared to intermediate-risk patients, as suggested by Armand and colleagues in cases of smaller cohorts [22].

As first an outcome-oriented approach for the lowest smoothed plot of the martingale residuals was performed to determine a cut point for WT1 expression at transplant and at relapse [24,25]. Martingale residuals can be used to assess the true functional form of a particular covariate [24] Briefly, due to an extremely skewed distribution of values, WT1 copy values were transformed using a natural log-transformation. WT1 was coded as an indicator variable in the Cox proportional hazards model. The Cox proportional hazards model is essentially a regression model commonly used statistically for investigating the association between the survival time of patients and one or more predictor variables. For distinct values of WT1 (≤300, ≤250, ≤200, ≤150, ≤100 and ≤50), we created an indicator variable and then fit the Cox model with this new covariate to obtain the log-likelihood. The value of WT1 that maximizes the log-likelihood determined the optimal cut point. The same approach was performed for the LDH level at relapse.

Next, Cox proportional hazard models were fitted to estimate hazard ratios. The hazard rate is the instantaneous rate of death (or failure) given that an individual has survived up to that time. The ratio between two groups’ hazard rates is a measure of the chance of an event in one group compared to that of another at a particular time or over a subset of a study’s time. Cox regression analysis was used to check whether variables such as disease stage at HSCT, DRI, WT1 at transplant, WT1 at relapse, white blood cells (WBCs) at relapse, BM blasts at relapse, platelet count at relapse, GVHD (acute and chronic) before relapse and LDH at time of relapse affected survival.

## 4. Results

### 4.1. Patient and Transplant Characteristics

The patient and transplant characteristics of the analyzed cohort are summarized in Table 1. Of note, the majority of patients had a high-risk disease. When stratifying the patients on the basis of the DRI [22], combining the disease risk at diagnosis and stage risk at transplant, 58.6% were assigned to a high or very high DRI.

All patients received a T-cell depleted graft. Graft-versus-host disease prophylaxis included cyclosporine (CSA) and methotrexate (MTX) in matched sibling donor transplants, CSA-MTX and rabbit anti-thymocyte globulin in unrelated donor transplants and tacrolimus–mycophenolate mofetil combined with post-transplant cyclophosphamide in case of haploidentical grafts.

### 4.2. Relapse and Treatment Characteristics

Detailed relapse and treatment characteristics of the present cohort are given in Table 2. The median time to relapse after allogeneic HSCT was 8.3 months (IQR: 5.7–16.1). Of note, at the time of relapse, 18 (62%) patients had still active immunosuppressive therapy due to GVHD, but tapering and successive suspension was possible before AZA/DLI initiation in all patients except one. As reported in Table 2, the median number of Azacytidine cycles was *n* = 2 (IQR: 1–24). A total of 11/29 (31%) patients were not able to receive DLI. The main reasons were the development of GVHD (*n* = 3), disease progression (*n* = 4) under first two AZA cycles, donor availability and, in one patient, bridging to a second allogeneic HSCT.

### 4.3. Safety and Toxicity

The median neutrophil count at time of relapse/AZA initiation was 915/mm^3^ (IQR: 30–3750/mm^3^), while the median platelet count was 52,000/mm^3^ (IQR: 13.000–248.000/mm^3^). Generally, treatment was well tolerated. Deviations from the programmed AZA schedule with temporary dose reductions to 50 mg/m^2^ for 7 days or 75 mg/m^2^ for 5 days were necessary in a total of four patients. Infectious complications were the main adverse events, namely pneumonia (*n* = 9, 31%), cholangitis (*n* = 1, 3.4%), tissue abscess (*n* = 4, 13.8%) or sepsis (*n* = 1, 3.4%). No patients died due to treatment toxicity. Of note, in 12 (41.3%) patients only an uncomplicated fever of unknown origin or no complications occurred. Interestingly, in 10 (34.5%) cases, the mentioned complications evolved under disease progression, so distinguishing between treatment-related toxicity or disease-related morbidity was difficult.

### 4.4. GVHD

Under AZA/DLI treatment, three patients developed GVHD, two with new onset and one with significant impairment, requiring immunosuppression treatment. One patient developed overall mild cGVHD involving skin and liver after 14 cycles of AZA with five DLIs. The second showed overall moderate cutaneous cGVHD after 10 AZA cycles with four DLIs, while the third developed overall mild gastrointestinal and skin cGVHD after a total of 15 AZA cycles with six DLIs. Interestingly, all three high-risk AML patients had an overall response to AZA/DLI, one with a stable disease lasting for 17.4 months and the latter two with complete remission lasting 18.9 and 28.2 months, respectively.

### 4.5. Treatment Response and Survival

Overall, after a median time to response of 4.9 (IQR: 2.4–29.7) months to AZA/DLI, seven patients (24.1%) achieved a complete remission (CR) and 5 = five patients (17.2%) a stable disease (SD), resulting in an overall response rate of 41.4%. The median duration of the response reached 11.9 (IQR: 1.6–17.4) months for SD patients and 19.5 (IQR: 11.5–29.7) months for CR patients. The median OS after AZA/DLI of the entire cohort marked 7.2 (IQR: 3.2–14.8) months. After a study duration of 37.5 months, 26 (89.7%) patients died due to disease progression or relapse, marking a median OS of 6.8 (IQR: 3.2–14.8) months (Figure S1). Of note, none of the patients receiving the reduced dosage of Azacytidine with 100 mg d1–5 responded, so currently, where possible, we aim to apply the standard dosage of 75 mg/m^2^ for 7 days. At time of last FU, 9 (31%) of them had a concomitant infection such as pneumonia, perianal abscess or fungoid sinusitis. Two further patients died in CR, of whom one due to COVID-19-related pneumonia (OS of 27.8 months) and one due to a colon perforation caused by CMV colitis (OS of 13.5 months). A third one died due to CNS and pulmonary aspergillosis during the second transplant, marking an OS of 47 days and representing the only case being able to proceed to a second transplant. In the OS analysis, no patient had to be censored because status (death) was known for all patients at the end of the observational period.

**Figure 1:**
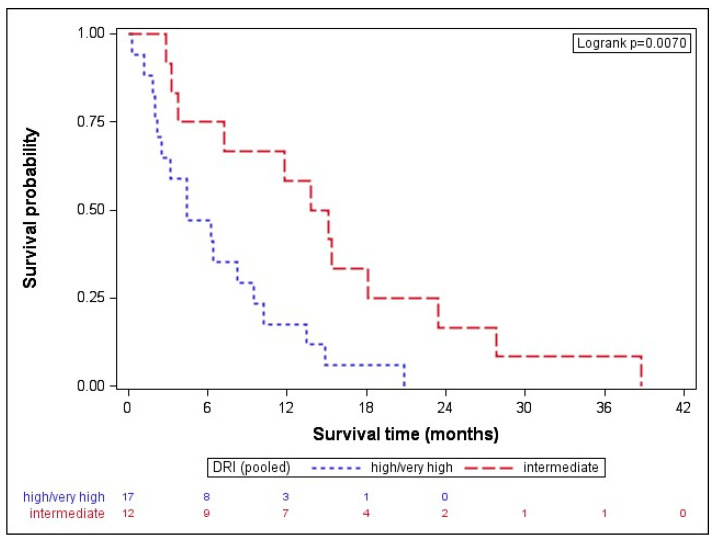
Overall survival after AZA/DLI is associated with DRI. The high-/very high-risk subgroup had a statistically significantly shorter OS with 4.4 (IQR: 2.2–9.5) months than the intermediate-risk patients with a median OS of 14.5 (IQR: 5.5–20.8) months, *p* = 0.007. DRI indicates disease-risk index.

When dividing the results by disease type, the median OS in AML was 8.0 (IQR: 2.4–15.1) months, while MDS patients had a median OS of and 7.2 (IQR: 3.3–13.8) months. While the disease type (*p* = 0.795) did not seem to impact on the outcome after AZA/DLI, the disease stage before transplant showed a clear trend. Patients in CR before transplant (*n* = 19) had a longer median OS with 11.8 (IQR: 2.8–18.1) months than patients with active disease (*n* = 10), formally SD or progressive disease (median OS of 6.4, IQR: 4.5–9.6 months, *p* = 0.061). Given that clear statistical significance failed, probably due to the small size of the subgroups, next, we pooled together very high and high DRI-scored patients and compared their OSs with intermediate DRI patients [22]. The high-/very high-risk subgroup had a statistically significantly shorter OS with 4.4 (IQR: 2.2–9.5) months than intermediate-risk patients with a median OS of 14.5 (IQR: 5.5–20.8) months, *p* = 0.007 (Figure 1).

### 4.6. WT1

**Figure 2:**
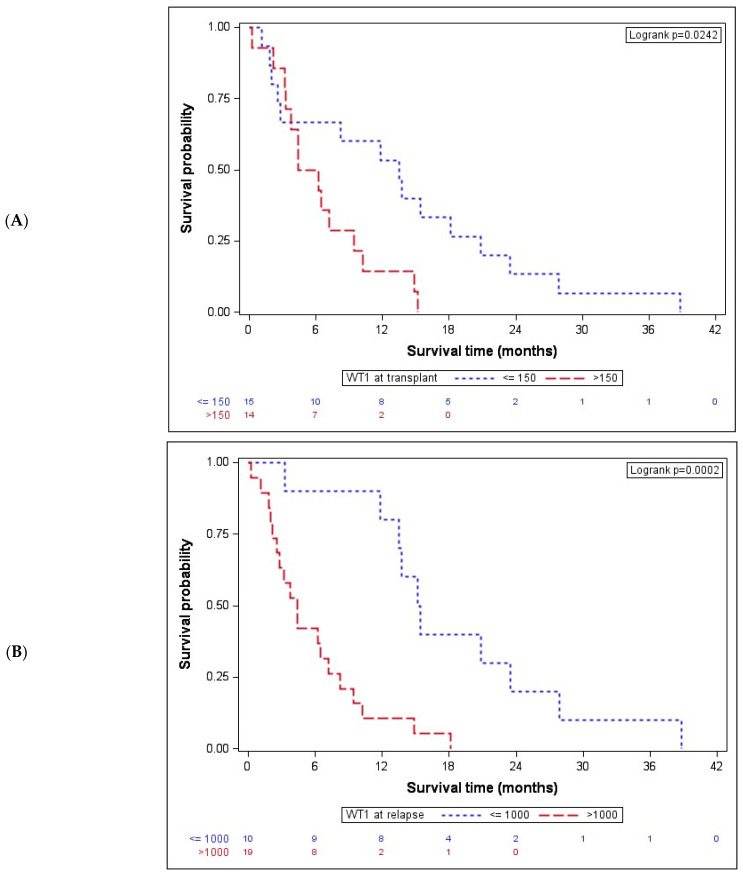
Bone marrow WT1 expression is associated with OS after AZA/DLI. (**A**) At the time of transplant, WT1-overexpressing patients (*n* = 14) had a significantly lower median OS after AZA/DLI of 5.3 (IQR: 3.3–9.5) months than patients with low WT1 expression (*n* = 15, ≤150 copies), with a median OS of 13.5 (IQR: 2.5–20.8) months, *p* = 0.024. (**B**) Patients (*n* = 10) with ≤1000 WT1 bone marrow copies at time of relapse had a significantly longer OS with 15.3 (IQR: 13.5–23.4) months than (*n* = 19) patients overexpressing WT1 and showing an OS of 4.4 (IQR: 2.2–8.3) months, *p* = 0.0002. WT1 indicates Wilms tumor gene 1.

After showing a significant association with the acknowledged clinical marker DRI, we next investigated the association of OS after AZA/DLI with molecular markers, namely BM WT1 expression. Previously, we reported, in a large AML cohort, that its overexpression after induction chemotherapy may represent an additional MRD tool for risk stratification in formally CR-classified patients [23]. In the present cohort, we first analyzed the association of BM WT1 expression at time of transplant with OS. Given that WT1 expression is a binary variable and an extremely skewed WT1 value distribution was evident, a natural log-transformation was performed in order to obtain a cut-off value for the stratification of patient subgroups. By applying the Cox proportional hazards model, a cut-off value of 150 copies (HR 2.6, CI 95 1.1–6.3) was obtained. In fact, at the time of transplant, WT1-overexpressing (>150 copies) patients had a significantly shorter median OS after AZA/DLI of 5.3 (IQR: 3.3–9.5) months than patients with low WT1 expression (≤150 copies), which was 13.5 (IQR: 2.5–20.8) months, *p* = 0.024 (Figure 2A).

Given that WT1 expression at transplant was shown to be associated with OS after AZA/DLI, next, WT1 expression at time of post-transplant relapse was analyzed. Again, the same model for the cut-off determination, which maximized the log-likelihood of the regression model with a cut-off value of WT1 ≤ 1000 copies (HR 5.4, CI95 2.0–14.2), was applied. In fact, dividing the patients on the basis of the WT1 copy number at relapse resulted highly significant in terms of OS. Patients (34.5%) with ≤1000 WT1 bone marrow copies at time of relapse had a significantly longer OS with 15.3 (IQR: 13.5–23.4) months than (65.5%) patients overexpressing WT1 (>1000), with an OS of 4.4 (IQR: 2.2–8.3) months, *p* = 0.0002 (Figure 2B).

In order to confirm these results, a Cox regression model was used. Possible predictors, such as the disease stage at HSCT, DRI, GVHD, WT1 at transplant and at relapse, WBC at relapse, BM blasts at relapse, platelet count at relapse and LDH at relapse, were analyzed via stepwise selection. WT1 expression at time of relapse and DRI were the strongest predictor for OS (Table 3).

In addition, the LDH level at time of relapse resulted statistically significant in association with OS after AZA/DLI. Using the same model as used for WT1 expression, a cut-off value of 390 U/l was determined for LDH (HR 2.37, CI95% 1.1–5.2), and patient subgroups were subsequently confronted. Indeed, patients (41.4%) with an enhanced LDH > 390 U/l at relapse had a statistically significantly shorter median OS of 3.8 months (IQR: 2.0–8.4) versus 11.8 months (IQR: 4.4–15.2) for patients (58.6%) with an LDH level ≤ 390 U/l, *p* = 0.029 (Table 3).

## 5. Discussion

Few effective salvage treatment regimens are available for patients who exhibit relapse of AML and MDS after allogeneic HSCT. Acquired multi-drug resistance [26] and increased gene methylation status may lead to the downregulation of immunogenic cell cycle checkpoint proteins [27,28]. Azacytidine increases the expression of immunogenetic antigens that can subsequently be recognized by donor lymphocytes [7]. In the present study, Azacytidine treatment was combined with DLI in patients with post-transplant AML/MDS relapse, leading to an overall response rate of 41,4%. In this particularly high-risk cohort, 24.1% of patients achieved CR, while 17.2% showed stable disease. The median duration of this response was 12.8 months, ranging from 11.5 to 29.7 months for CR patients. These response rates are in line with previously published data for AZA/DLI in the same setting. Czibere and colleagues reported, in a multicenter retrospective analysis, a CR rate of 23% for 22 AML/MDS patients [5]. Two large studies have confirmed non-complex cytogenetics and low–intermediate genetic risk profiles as significant factors associated with OS [2,29]. In our study, the DRI, which incorporates initial cytogenetics and the disease stage at transplant, was significantly associated with OS, and patients with high and very high DRI had as significantly shorter OS after AZA/DLI than patients with intermediate DRI. Of note, the DRI maintained its statistical significance even after a stepwise selection of the analyzed predictors in a Cox regression analysis.

According to these findings, the hypothesis arises whether MRD-positive hematologically CR high-risk patients may benefit from more aggressive treatments, such as intensification of the conditioning regimen, early discontinuation of immunosuppression and/or DLI based regimens. Wilms tumor 1 gene was proposed as a potential candidate for MRD in AML by several study groups [30,31,32,33]. We have previously shown in a large AML cohort that WT1 overexpression was significantly associated with impaired outcome after intensive induction chemotherapy in formally CR achieving patients [23]. Bone marrow WT1 expression at two significant decisive time points, namely immediately after first induction chemotherapy and before allogeneic HSCT, showed statistically significant correlation with OS and PFS [23]. Similar results have been reported by Valkova and colleagues, as they and others have investigated the role of WT1 expression not only in the bone marrow but also in peripheral blood [31,32,33]. Also, Rautenberg and colleagues proposed peripheral blood WT1 expression as a pre-transplant MRD marker, predicting post-transplant outcome and, hence, improving peri-transplant management in high-risk AML/MDS patients [34].

In the present analysis, we confirm the previously published bone marrow WT1 cut-off value of 150 copies at transplant [23] in the present cohort also. Patients with >150 WT1 copies at transplant receiving AZA/DLI treatment for post-transplant relapse had a significantly shorter OS than patients with ≤150 copies. In the present cohort, in addition, bone marrow WT1 expression was analyzed in all patients at the time of relapse. As for WT1 expression at time of transplant, the same statistical method was applied in our present cohort, and a cut-off value of 1000 copies obtained for WT1 expression at the time of post-transplant relapse. In fact, WT1 expression of 1000 copies was significantly associated with OS; patients with >1000 WT1 copies at relapse had a significantly shorter OS after AZA/DLI than patients with ≤1000 copies. The fact that the pre-transplant cut-off is significantly lower may be related to its MRD character at time of transplant compared to the high value at post-transplant relapse with mostly active disease.

To our best knowledge, this is the first study to describe this association in this setting. Currently, there is no standardization as to when exactly WT1 expression should be performed or which cut-off value should generally be considered as clinically significant in this setting.

Given this strong association of WT1 expression with disease activity, recently, Chapius and colleagues targeted WT1 using T-cell receptor gene therapy in the post-transplant setting [35], while Augsberger and colleagues showed that WT1-bound-T-cell bispecific (TCB) antibodies facilitate the potent killing of AML cells in vitro [36]. Further insights in this field will help us to understand whether WT1 will have a potential role not only as an MRD marker but also as a treatment target in AML and MDS.

In the study of Claiborne and colleagues [37], molecular/cytogenetic-only relapse, the development of cGVHD after therapy initiation and the number of treatment cycles were associated with a significantly higher response rate to AZA/DLI. Interestingly, in our cohort, three patients developed cGVHD after AZA/DLI treatment and showed a complete response of their AML, with a median overall survival of 18.4 months. The association of treatment response and GVHD underlines the link to the graft-versus-leukemia (GVL) effect. GVHD is triggered by donor T cells, which respond to recipient tissue antigens secondary to mismatches between major and/or minor histocompatibility antigens. Minor HLA antigens restricted to the hematopoietic system may be able to enhance GVL responses, while more broadly expressed minor HLA antigens contribute to both GVHD and GVL [38]. One of these CR patients developing GVHD was the only AML patient in our cohort relapsing, only molecularly, with the appearance of his complex karyotype. The high quality of his response to AZA/DLI with a nearly 2-year OS indicates that low disease burden may be more susceptible to T-cell-mediated immune responses. He maintained his complete response after a total of 10 AZA cycles and four DLIs with an OS of 20.8 months.

The significant association of better response to AZA/DLI with only molecular/cytogenetic relapse was reported previously [37]. In our cohort, only one patient relapsed cytogenetically and showed an excellent response to AZA/DLI. The published data suggest that pre-emptive DLI may have a role in cytological MDS and AML relapses, particularly in T-cell-depleted and non-myeloablative HSCT [39,40]. In the same context, Rautenberg and colleagues have recently shown, for a similar cohort size (*n* = 35), that combining WT1 expression-based MRD monitoring and pre-emptive therapy with hypomethylating agents (HMA) and DLI represents a feasible and effective approach. They applied DLI even after every cycle and could demonstrate a CR rate of 37% [41].

Giullaume and colleagues reported promising results in 30 high-risk AML patients, who received maintenance with 12 cycles of low-dose Azacytidine with DLI [42]. Even they report a suspension of DLI in nearly half of their patients (*n* = 13) and discontinuation of AZA in the majority of their patients (*n* = 20), primarily due to GVHD appearance, underlining clearly the fragility of this patient subset. In our study, 37.9% (*n* = 11) of patients were not able to receive DLI, mainly due to disease progression, GVHD appearance and donor availability. Given that 37.9% of the patients did not arrive to receive DLI after AZA treatment, the separation of this subgroup in the analysis may be formally requested. As in the majority of published evidence, our cohort size reflects a real-world experience in terms of a single-center experience in this poor-risk fragile cohort. Unfortunately, our study has several limiting factors, namely the retrospective character, the lack of a control group for the comparison of the drug effect and the small cohort size.

## 6. Conclusions

In conclusion, the association of bone marrow WT1 expression, at transplant and at time of relapse, with the overall survival supports the use of WT1 as an effective MRD marker. Additional multicenter prospective clinical trials are mandatory to validate whether WT1 overexpression at decisive treatment time points may guide early treatment initiation in patients, nowadays considered in CR. In the current era of available targeted drugs, such as FLT3- and IDH-inhibitors, especially in combination with venetoclax and hypomethylating agents with and without DLI, the role of WT1 may be considered a useful tool warranting deeper investigation. Initial phase II approaches of HMA/DLI with ruxolitinib [43] and with lenalidomide [44] in this setting are currently emerging.

## Figures and Tables

**Table 1:** Patient and transplant characteristics. *n* indicates number; HSCT, hematopoietic stem cell transplant; AML, acute myeloid leukemia; MDS, myelodysplastic syndrome; MPS, myeloproliferative syndrome; CR, complete remission; SD, stable disease; MAC, myeloablative conditioning; RIC, reduced intensity conditioning; MSD, matched sibling donor; MUD, matched unrelated donor; Haplo, haplo-identical donor; PBSC, peripheral blood stem cell; BM, bone marrow.

Variable	*n*=	%/IQR
Patients	29	
Median age at HSCT, years	52.3	25–68
Diagnosis before HSCT		
AML	20	69
MDS	9	31
Cytogenetic/molecular risk stratification		
Adverse	17	58.6
Intermediate	9	31
Missing	3	10.3
Disease Risk Index stratification		
Intermediate	12	41.4
High	11	37.9
Very high	6	20.7
Reason for high-risk stratification		
Primary refractory	9	31
Relapse	7	24.1
t-AML or post-MDS/MPS	4	13.8
Extramedullary disease	1	3.4
Disease status at HSCT		
CR1/>CR1	19	65.5
SD	3	10.3
Relapse/progressive	7	24.1
Conditioning regimen		
MAC (sequential)	25 (5)	86.2 (17.2)
RIC	4	13.8
Donor type		
MSD	7	24.1
MUD	16	55.2
Haploidentical	6	20.7
Graft source		
PBSC	21	72.4
BM	8	27.6
WT1 at transplant, range copies	184	15–7335

**Table 2:** Relapse and treatment characteristics. *n* indicates number; HSCT, hematopoietic stem cell transplant; AML, acute myeloid leukemia; MDS, myelodysplastic syndrome; BM, bone marrow; WT1, Wilms tumor gene 1; aGVHD, acute graft versus host disease; cGVHD, chronic graft versus host disease; AZA, Azacytidine; DLI, donor lymphocyte infusions; CR, complete response; PD, progressive disease.

Variable	*n*=	%/IQR
Median time to relapse, months	8.3	5.7–16.1
Diagnosis at relapse		
AML	24	82.8
MDS	5	17.2
BM chimerism at relapse, median	85.5	0–99%
*WT1* at relapse, median copies	1570	89–10665
aGVHD before relapse		
Grade I	6	20.7
Grade II	9	31.0
Grade III	1	3.4
cGVHD before relapse		
Mild	4	13.8
Moderate	6	20.7
Severe	1	3.4
Immunosuppression at relapse	18	62.1
Taper/stop before AZA start	17	58.6
Number of AZA cycles, median	2	1–24
1–3 cycle	13	44.8
4–6 cycles	8	27.6
>6 cycles	8	27.6
Number of DLI, median	1	0–6
no DLI	11	37.9
1–3 DLI	14	48.3
4–6 DLI	4	13.7
GVHD post AZA/DLI	3	10.3
Response to AZA/DLI		
CR	7	24.1
SD	5	17.2
PD	17	58.6
Duration of response, months		
CR	19.5	11.5–29.7
SD	11.9	1.6–17.4

**Table 3:** Overall survival results and potential predictors for survival. DRI high-/very high-risk with shorter OS of 4.4 months than DRI intermediate-risk patients, with 14.5 months, *p* = 0.007. WT1 overexpression at transplant correlated with median OS of 5.3 versus 13.5 months, *p* = 0.0242. Patients with ≤1000 WT1 copies at time of relapse had longer median OS of 15.3 versus 4.4 months, *p* = 0.0002. Patients with an enhanced LDH >390 U/l at relapse had a shorter median OS of 3.8 versus 11.8 months of patients with low LDH, *p* = 0.0286. Univariate analyses show outcomes in DRI, WT1 at transplant, WT1 at relapse and LDH at relapse. Cox regression analyses were used to identify which potential predictors affect the survival. A stepwise selection was performed. WT1 expression at time of relapse was selected as the strongest predictor for OS when including relapse variables. When regarding only pre-relapse variables, the DRI was the strongest predictor for OS.

	Survival Analysis		Univariate Model		
	Median (IQR)	*p* *		HR (95% CI)	*p* **
DRI high/very high intermediate	4.44 (2.20–9.47) 14.47 (5.51–20.78)	0.0070	high/very high vs. intermediate	3.05 (1.31; 7.11)	0.0096
WT1 at transplant ≤150 >150	13.48 (2.53–20.81) 5.33 (3.29–9.47)	0.0242	>150 vs. ≤150	2.65 (1.11; 6.34)	0.0289
WT1 at relapse ≤1000 >1000	15.27 (13.48–23.44) 4.41 (2.20–8.25)	0.0002	>1000 vs. ≤1000	5.39 (2.04; 14.24)	0.0007
LDH at relapse ≤390 >390	11.84 (4.44–15.16) 3.79 (2.01–8.35)	0.0286	>390 vs. ≤390	2.37 (1.07; 5.25)	0.0335

* log-rank test; ** Cox regression model.

## Data Availability

The raw data supporting the conclusions of this article will be made available by the authors upon request.

## Supplementary Materials

The following supporting information can be downloaded at: https://www.mdpi.com/article/10.3390/cancers16173070/s1, Figure S1: Overall 6-, 12-, 18- and 24-month survival rates with 95% confidence intervals.
