# Supplementary material for: WT1 Expression Is Associated with Poor Overall Survival after Azacytidine and DLI in a Cohort of Adult AML and MDS Patients
Source: Cancers (Basel). 2024 Sep 4;16(17):3070. doi: 10.3390/cancers16173070 (PMC11394520; doi:10.3390/cancers16173070)
Supplement: Supplementary file 1 [file cancers-16-03070-s001.zip › cancers-3083645-supplementary.pdf]

Supplementary Figure S1. Overall 6-, 12-, 18- and 24-months survival rates with 95%-confidence intervals.

A

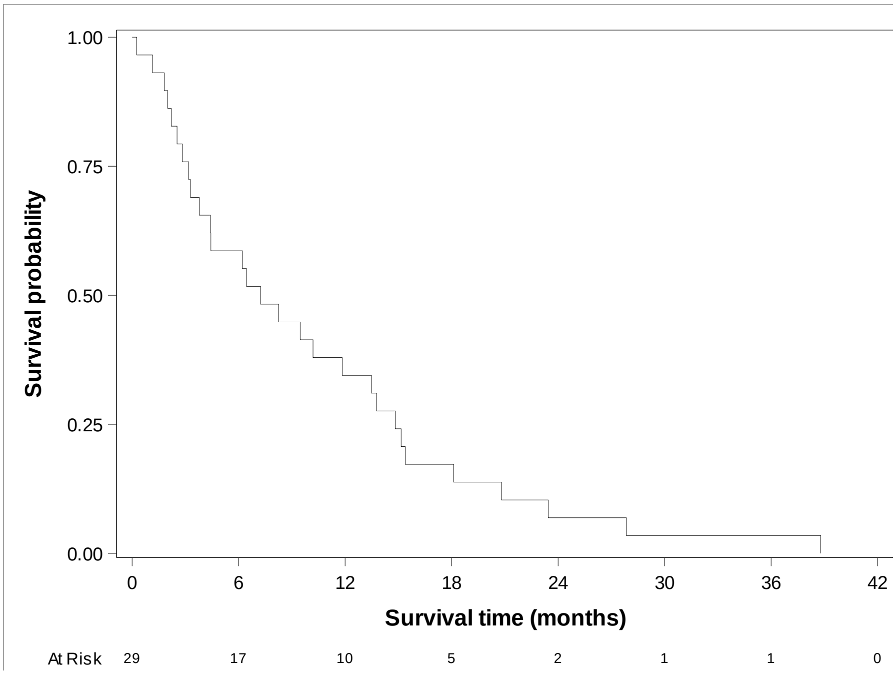

B

time point (months)	Survival rate (%)	95% CI
6	58.6	40.7 - 76.6
12	34.5	17.2 - 51.8
18	17.2	3.5 – 31.0
24	6.9	2.3 – 16.1
